# Circunferência da Cintura, uma Medida Simples para a Obesidade Infantil?

**DOI:** 10.36660/abc.20200031

**Published:** 2020-04-06

**Authors:** Luciana Nicolau Aranha, Gláucia Maria Moraes de Oliveira

**Affiliations:** 1 Universidade Federal do Rio de JaneiroRio de JaneiroRJBrasilUniversidade Federal do Rio de Janeiro, Rio de Janeiro, RJ - Brasil

**Keywords:** Criança, Adolescente, Obesidade Infantil, Sobrepeso, Fatores de Risco, Pesos e Medidas Corporais, Pressão Arterial

A obesidade infantil continua aumentando em todas as regiões do mundo, sendo considerada um dos grandes desafios de saúde pública. A prevalência aumentou de menos de 1% em 1975 para 5,6% em meninas e 7,8% em meninos em 2016.^1^ No Brasil, os dados também são preocupantes, uma vez que no último levantamento oficial realizado pelo Instituto Brasileiro de Geografia e Estatística (IBGE), constatou-se que entre 2008 e 2009, 51,4% dos meninos e 43,8% das meninas com idade entre 5 a 9 anos apresentavam sobrepeso ou obesidade.^2^

Crianças e adolescentes com obesidade têm cinco vezes mais chances de serem obesos quando adultos.^3^ Além do mais, a obesidade na infância está associada com a elevação da pressão arterial, resistência à insulina, diabetes mellitus, dislipidemia e com o aumento da morbimortalidade cardiovascular na idade adulta.^4^ Por isso, é importante identificar o excesso de gordura corporal nesta população e criar estratégias para prevenir o desenvolvimento de doenças crônicas no futuro.

Com o objetivo de detectar crianças e adolescentes com risco cardiometabólico, sugeriu-se o uso de indicadores antropométricos como ferramentas de triagem epidemiológica, uma vez que são métodos não invasivos, de baixo custo e de fácil aplicação.^5,6^ A circunferência da cintura (CC) por exemplo, é um indicador de adiposidade central relacionada a complicações metabólicas da obesidade na população pediátrica.^7,8^ Porém, ainda não existem pontos de corte de CC padronizados para classificação de adiposidade abdominal em crianças e adolescentes, o que torna o seu uso limitado.

Estudos descrevendo valores de percentis para CC têm apresentado resultados diferentes, uma vez que os valores de CC podem ser influenciados por idade, sexo e grupos étnicos,^9-11^ dificultando o estabelecimento de valores de referência globais para essa medida antropométrica.

Na edição atual dos Arquivos Brasileiros de Cardiologia, Santos et al.,^12^ publicaram estudo longitudinal, realizado com 22.000 crianças (11.199 meninos) com idades entre 6 e 10 anos de idades, matriculadas em escolas públicas e particulares de 13 cidades do estado de São Paulo. Os autores apresentaram curvas de referência da CC específicas para idade e sexo e pontos de corte para identificar crianças com risco de obesidade. Os autores descreveram que aproximadamente 30% das crianças apresentaram excesso de gordura, sendo classificados com sobrepeso ou obesidade, conforme o índice de massa corporal. As análises da curva ROC mostraram o percentil 75 como ponto de corte ideal para risco de sobrepeso e obesidade e que a obesidade é claramente diagnosticada nas crianças com a CC classificada a partir do percentil 85.^12^

Ao comparar as curvas da CC (percentil 50) com os resultados de um estudo brasileiro realizado com 2919 escolares com idade entre 7 a 10 anos em 2007,^13^ na cidade de Florianópolis, Santos et al.,^12^ observaram que as curvas atuais de percentis foram mais elevadas, chegando a um aumento de 4,0 cm nas meninas aos 10 anos de idade. Diferenças metodológicas podem explicar estes valores discrepantes, embora os autores de ambos os estudos utilizaram a mesma metodologia para a aferição da CC. No entanto, a população brasileira tem um alto grau de miscigenação e, como citado acima, os valores de CC podem ser influenciados pela etnia, o que poderia explicar a diferença desses resultados.

Como mencionado, poucos estudos no Brasil descreveram valores de ponto de corte da CC em uma grande amostra com vários grupos etários, o que justifica a importância dessas investigações para a literatura científica. Porém, como os próprios autores relataram como limitação, os valores das curvas de percentis foram determinados com base em uma amostra de crianças do estado de São Paulo, sendo aconselhável para a generalização dos resultados, utilizar amostras representativas de todos os estados do Brasil. Além disso, os valores propostos precisarão ser validados em outra população com características semelhantes.

Recentemente, Xi et al.,^14^ propuseram pontos de cortes internacionais de CC específicos por idade e sexo para definir obesidade central, com base em dados de 113.453 crianças e adolescentes, com idades entre 4 e 20 anos, de oito países de diferentes regiões: Bulgária, China, Irã, Coréia, Malásia, Polônia, Seychelles e Suíça. Os autores estabeleceram o percentil 90 como ponto de corte para identificar a obesidade central nessa população, e verificaram que esse percentil em crianças com peso normal teve um bom desempenho para prever o risco cardiovascular, sugerindo que possa ser útil para avaliar a adiposidade abdominal em crianças e adolescentes em diferentes países.
