# Supplementary material for: Circunferência da Cintura, uma Medida Simples para a Obesidade Infantil?
Source: Arq Bras Cardiol. 2020 Apr 6;114(3):538–9. [Article in Portuguese] doi: 10.36660/abc.20200031 (PMC7792731; doi:10.36660/abc.20200031)
Supplement: Translation

# Translation: Waist Circumference: A Simple Measure for Childhood Obesity?

**Authors:** Luciana Nicolau Aranha, Gláucia Maria Moraes de Oliveira

**Affiliations:** 1 BrazilUniversidade Federal do Rio de Janeiro, Rio de Janeiro, RJ - Brazil

**Keywords:** Child, Adolescent, Pediatric Obesity, Overweight, Risk Factors, Body Weights and Measures

Childhood obesity has been increasing all over the world and considered one of the major challenges in public health. The prevalence increased from less than 1% in 1975 to 5.6% and 7.8% among girls and boys, respectively, in 2016.^1^ No less worrying are the recent data from the Brazilian Institute of Geography and Statistics (IBGE) showing that in 2008-2009, 51.4% of boys and 43.8% of girls aged between 5 and 9 years were overweight or obese.^2^

Obese children and adolescents are five times more likely to become obese adults.^3^ Besides, childhood obesity has been associated with hypertension, insulin resistance, diabetes mellitus, dyslipidemia, and increased morbidity and mortality in adult life.^4^ Therefore, it is important to screen for excess body fat in this population and create strategies to prevent the development of chronic diseases in the future.

Anthropometric indicators have been suggested as epidemiological screening tools to detect children and adolescents with high cardiometabolic risk, because of their non-invasiveness, low cost, and easy application.^5,6^ Waist circumference (WC), for example, is an indicator of central adiposity associated with metabolic complications of obesity in the pediatric population.^7,8^ However, the cut-off points of WC for classification of abdominal adiposity in children and adolescents have not been established yet, which limits its use.

Studies describing percentile values of WC have reported different results; WC measurements may be affected by age, sex and ethnicity,^9-11^ which makes the establishment of global reference values difficult.

In the current issue of Arquivos Brasileiros de Cardiologia, Santos et al.^12^ published a longitudinal study with 22,000 children (11,199 boys) aged between 6 and 10 years, attending public and private schools of 13 cities in Sao Paulo state. The authors presented WC reference curves for age and sex and cut-off points to identify children at risk for obesity. The authors described that approximately 30% of the children had excess body fat and classified as overweight according to body mass index. ROC curve analysis revealed that the 75th percentile was the optimal cut-off point for overweight and obesity, and that obesity was easily diagnosed among children with WC values above the 85th percentile.^12^

When the WC curves (50th percentile) were compared with results of another Brazilian study with 2,919 students aged 7 to 10 years carried out in Florianopolis, Brazil, in 2007,^13^ Santos et al.^12^ observed that the current percentile curves are higher, with an increase of up to 4.0 cm among girls at the age of 10. These discrepancies may be explained by methodological differences, although the method used for WC measurements was the same in both studies. However, it is known that the Brazilian population is highly mixed and, as above mentioned, WC can be influenced by ethnicity, which may explain the difference between the results.

Again, few studies have addressed cut-off values for WC in a large population including different ethnical groups in Brazil, which reinforces the importance of these investigations to contribute to the scientific literature. However, as the authors mentioned as limitation of the study, the percentiles curves were established based a sample of children in the State of Sao Paulo, and hence it is advisable that representative samples of all geographic regions of the country be studies for generalization of results. Besides, the values proposed need to be validated in other populations with similar characteristics.

Recently, Xi et al.^14^ proposed international WC percentile cut-off points, specific for age and sex, to define central obesity based on data of 113,453 children and adolescents aged 4-20 years from eight countries in different regions (Bulgaria, China, Iran, Korea, Malaysia, Poland, Seychelles, and Switzerland). The 90th percentile was established as WC cut-offs to detect central obesity in this population, with good performance in predicting cardiovascular risk in normal weight children and was suggested to be used in the assessment of abdominal adiposity in children and adolescents in different countries.
